# Fatigue crack closure: a review of the physical phenomena

**DOI:** 10.1111/ffe.12578

**Published:** 2017-02-01

**Authors:** R. Pippan, A. Hohenwarter

**Affiliations:** ^1^ Erich Schmid Institute of Materials ScienceAustrian Academy of SciencesLeoben8700Austria; ^2^ Department of Materials PhysicsMontanuniversität LeobenLeoben8700Austria

**Keywords:** fatigue crack growth, oxide‐induced crack closure, plasticity‐induced crack closure, roughness‐induced crack closure, threshold

## Abstract

Plasticity‐induced, roughness‐induced and oxide‐induced crack closures are reviewed. Special attention is devoted to the physical origin, the consequences for the experimental determination and the prediction of the effective crack driving force for fatigue crack propagation. Plasticity‐induced crack closure under plane stress and plane strain conditions require, in principle, a different explanation; however, both types are predictable. This is even the case in the transition region from the plane strain to the plane stress state and all types of loading conditions including constant and variable amplitude loading, the short crack case or the transition from small‐scale to large‐scale yielding. In contrast, the prediction of roughness‐induced and oxide‐induced closures is not as straightforward.

**Nomenclature**
Δ*CTOD*: = cyclic crack tip opening displacement
Δ*r*_*pl*_: = cyclic plastic zone size
*d*_*n*_: = dimensionless factor describing hardening behaviour and stress state
*u*_*i*_: = displacements into the principal directions with *i* = 1,2,3
*d*: = distance between dislocations
Δ*K*_*eff*_: = effective stress intensity range
Δ*σ*_*eff*_ or Δ*ε*_*eff*_: = effective stress or strain range
*ε*_*y*_: = elastic strain contribution
Δ*ε*: = entire strain range
*n*: = hardening exponent
*b*: = magnitude of burgers vector
*x*_min_: = minimum width of a wedge
*r*_*pl*_: = monotonic plastic zone size
*x*_*i*_: = principal directions with *i* = 1,2,3
*α*: = rotation angle
*Κ*_max_: = stress intensity at maximum load
*K*_min_: = stress intensity at minimum load
*K*: = stress intensity factor
Δ*K*: = stress intensity factor range
*K*_*cl*_: = stress intensity factor when first contact between crack flanks occurs
*K*_*op*_: = stress intensity factor when last crack flank contact opens
*R*: = stress ratio (=*K*_min_/*K*_max_)
Δ*K*_*eff,th*_: = threshold of effective stress intensity factor range
*σ*_*y*_: = yield stress
*E*: = Young's modulus

## Introduction

In order to understand the fatigue crack propagation behaviour of materials as a function of loading condition, for instance, stress or strain amplitude, stress ratio, load history, environment, mixed mode loading, large‐scale or small‐scale yieldings and so on, it is very helpful to separate the different mechanisms affecting the crack propagation rate into intrinsic and extrinsic mechanisms.^1^

The intrinsic mechanisms cause the formation of new fracture surfaces at the crack tip. They are controlled by the monotonic and cyclic strains applied to the volume elements at and in front of the crack tip. For cyclic loading significantly below the fracture toughness, the cyclic deformation is the dominant part responsible for fatigue crack propagation. In fatigue of ductile metals, the crack extension per cycle is usually caused by plastic blunting and re‐sharpening of the crack tip,^2^, ^3^, ^4^, ^5^, ^6^, ^7^, ^8^, ^9^, ^10^ except for loads near the fracture toughness where static fracture or monotonically induced damage ahead of the crack tip can contribute significantly to the crack extension in each cycle. The contribution of monotonic loading‐induced fracture is more important in brittle materials such as intermetallic alloys, ceramics and some cast alloys.

The extrinsic mechanisms affect the monotonic and cyclic deformation at the crack tip and as a consequence influence the crack growth rate. The mechanisms that decrease or increase the deformation can have a shielding or anti‐shielding effect, respectively. Ritchie^1^ and Suresh^11^ classified the different extrinsic mechanisms into geometric, zone and contact shielding mechanisms and combined zone and contact shielding mechanisms. The geometric mechanisms, such as crack deflection or branching, are a consequence of the deviation of the real crack shape from the ideal plane crack with a straight crack front. They induce a local variation of the stress and strain fields at the crack along the crack front during crack propagation, which can be expressed by a local variation of the crack driving force and a mixing of the local loading modes. The zone shielding effects, for example, phase transformation, plasticity or residual stresses, also change the stress and strain fields, but can induce fracture surface contact during cyclic loading as well, similar to the other contact mechanisms.

The essence of the contact shielding mechanisms is a load transfer between the crack faces reducing the monotonic as well as the cyclic deformation at the crack tip during one load cycle compared with cracks where such load transfer is not active. Therefore, contact shielding mechanisms by corrosion debris‐induced closure (often denoted as oxide‐induced crack closure), roughness‐induced closure, crack bridging by ligaments or fibres, sliding crack surface interference and the combined zone and contact shielding mechanisms by plasticity‐induced crack closure and phase transformation‐induced crack closure are essential for the understanding of the fatigue crack propagation phenomena. Crack bridging by ligaments and fibres is important especially in brittle materials and composites, respectively,^12^, ^13^, ^14^, ^15^, ^16^, ^17^, ^18^ whereas sliding crack surface interference is more important under mixed mode loading.^1^, ^19^ These two mechanisms can induce a load transfer during the whole load amplitude, therefore reducing the monotonic and the cyclic deformation of the crack tip. On the other hand, crack closure induced by plasticity, roughness and corrosion debris causes a load transfer only during a certain part near the minimum load of the load amplitude and therefore only affects the cyclic deformation of the crack tip. In order to make the consideration easier, the present paper focuses only on the effect of these closure mechanisms. Despite the vast number of papers dealing with these mechanisms and their origin, the measurability and predictability, as well as their dependence on loading history, microstructure or crack length, there are several essential discrepancies regarding their interpretation and importance in the fatigue community.

The present paper is not thought to be a summary of about 10 000 papers dealing with this subject but is more intended to be a critical review dealing with the origin of crack closure, the measurability and predictability of the closure itself as well as its importance in lifetime simulation and the open questions in the different areas. It should be pointed out that this review concentrates on the phenomena of crack closure exclusively for external Mode I loading, because for Modes II and III, the sliding contacts (which reduce strongly the effective driving force) act during the whole load amplitude and the driving force cannot be simply determined by *K*_max_−*K*_*Cl*_.

The paper will start with a short historical overview, drawing then special attention to the physical origin of the fracture surface contacts in the tensile part of the loading, moving to the effect of contact forces on the crack stress field and discussing shortly the measurability, the predictability and the important parameters affecting crack closure under small‐scale yielding condition. Finally, the effect of closure under large‐scale or full‐scale yielding, which is typical for low cycle fatigue, will be considered.

### Historical background

The first observations on early crack initiation and the following fatigue crack propagation during cyclic loading were published by Ewing and Humphrey.^20^, ^21^ For a long time, fatigue crack growth investigations had been performed solely by optical microscopes. The development of the electron microscopes in the 1950s helped to discover special features such as striations, which indicate the cycle per cycle nature of crack growth.^22^ The application of the fracture mechanics started with the use of the stress intensity factor range to describe fatigue crack propagation by Paris^23^ in the beginning of the 1960s. Rice introduced a simple transformation scheme^24^ and suggested that the cyclic deformation in front of a crack could be described by the same equations as in the case of monotonic loading. One has only to exchange the stress intensity factor *K* by the stress intensity factor range Δ*K* = *K*_max_−*K*_min_, and the yield stress becomes twice the monotonic yield stress. Hence, the cyclic plastic zone, the cyclic plastic strain in front of the crack tip and the cyclic crack tip opening displacement is only a function of the Δ*K* and the cyclic plastic properties of the material. At the same time, the first damage^24^ and geometrical deformation models^2^, ^3^, ^4^, ^5^ were proposed to explain the fatigue crack propagation behaviour.

In 1970, Elber^25^, ^26^ discovered that a contact between the fracture surfaces could take place even during cyclic tensile loading. He attributed the occurrence of this fracture surface contact to the plastic deformation in the wake of a growing crack. This mechanism is nowadays usually called plasticity‐induced crack closure. During the following decade, several other mechanisms responsible for premature contact of the fracture surface were proposed: roughness‐induced crack closure,^27^, ^28^, ^29^, ^30^, ^31^ oxide‐induced crack closure or often termed corrosion debris‐induced crack closure^32^, ^33^, ^34^, ^35^, ^36^, ^37^ and phase transformation‐induced crack closure.^37^, ^38^, ^39^

A further important finding in the 1970s was the so‐called short crack effect,^40^, ^41^, ^42^, ^43^ which means that at the same driving force, Δ*K*, short cracks grow faster than long cracks, and short cracks can propagate even below the Δ*K*_*th*_ of a long crack. In the 1980s, it was shown that it was very helpful to subdivide the short crack phenomena into different types of short cracks, namely, microstructurally short, mechanically short, physically short and chemically short cracks.^11^, ^41^

Most of the studies in the last four decades used the crack closure effect as a key to understand the effects of load ratio and load interaction, the behaviour of physically short cracks and many of the microstructural effects on fatigue crack propagation. Despite the huge number of studies supporting the importance of crack closure, there are several papers that noticed doubts on this concept; see, for example, Refs. [37, 44, 45].

In particular, plasticity‐induced crack closure under plane strain loading conditions has been mistrusted, along with roughness‐induced and debris‐induced crack closures. The criticisms were most clearly pointed out in a paper of Louat *et al.* with the following statement^44^:

Plasticity originating from the crack tip does not induce crack closure and closure arising from asperity ridges due to oxides, corrosion products or surface roughness is small and insignificant unless the crack is completely packed with asperities.

Despite some intense discussions^45^, ^46^, ^47^, ^48^, ^49^ and attempts of clarification,^50^, ^51^, ^52^, ^53^, ^54^ some discrepancies regarding the importance of crack closure seem to be unsolved. The basic questions are as follows:

- Can *da*/*dN* be described solely by Δ*K*_*eff*_ (or another effective driving force, for example, Δ*J*_*eff*_) and
- Can Δ*K*_*eff*_ be simply calculated by

(1) $$\Delta K_{\mathrm{eff}}=K_{\max}-K_{\mathrm{cl}}$$

where *K*_max_ is the maximum of the stress intensity factor and *K*_*cl*_ is the stress intensity factor where the first fracture surface contact takes place.

This review will try to shed light onto some of these discrepancies and will point out a few important open questions.

## Closure Mechanisms

In this chapter, the physical origin of plasticity‐induced, roughness‐induced and oxide‐induced crack closures will be discussed. As mentioned in the Introduction, they are especially important in ductile materials. Ligament and fibre bridging as well as sliding crack surface interference will not be considered here. In the latter case, Δ*K*_*eff*_ can definitely not be determined by Eq. (1), because there exists no defined *K*_*cl*_. For the determination of the effective Δ*K*, the load transfer between the crack flanks at *K*_min_ and *K*_max_ would have to be known. In this case, a possible method to determine experimentally the effective Δ*K* would be given by an accurate analysis of the near crack tip opening displacement.^13^ Because of the 3D shape of the crack, and when additionally plasticity is involved, this analysis can be quite difficult and is not in focus of this paper.

### Plasticity‐induced crack closure

For the explanation of the plasticity‐induced crack closure, one has to distinguish between plane strain and plane stress case. For the plasticity‐induced crack closure under plane stress condition, the explanation is simple, and it is well accepted in the fatigue community.

**Figure 1:**
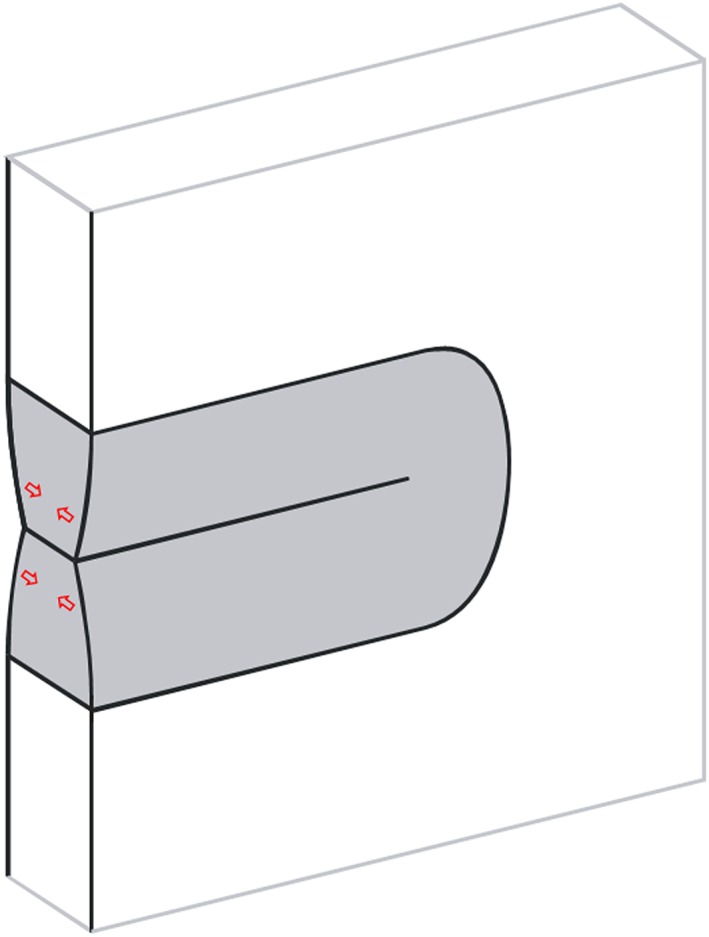
Illustration of the origin of the plasticity‐induced crack closure under plane stress conditions. The out‐of‐plane flow of the material in the plastic zone induces an elongation of the volume elements. The effect of the plastic wake on the cyclic plastic deformation can be interpreted as a wedge filled into the crack. [Colour figure can be viewed at wileyonlinelibrary.com]

Under plane stress conditions, the volume elements in the plastic zone are elongated. This elongation is mainly balanced by an out of the plane flow of the material – that is, the thickness within the plastic zone is reduced, as schematically illustrated in Fig. 1. When the crack propagates through the plastic zone, the stretched plastic wake comes into contact in the tension phase of the load amplitude.

Hence, the plasticity‐induced crack closure under plane stress conditions can be expressed as a consequence of an extra material layer behind the crack tip, which can be considered as a wedge that is inserted in the crack and reduces the cyclic plastic deformation at the crack tip and hence the fatigue crack growth rate. The formation of this plastic wedge is properly described by the Dugdale^55^ model worked out by Budiansky and Hutchinson,^56^ Führing and Seeger^57^ and by the mostly used modified versions of Newman.^58^ These strip yield models are well established to describe the stress ratio effects, load interaction effects or short crack effects.^59^, ^60^, ^61^ However, the disadvantage is that these models can only describe physically correct closure for the plane stress case. Newman^62^ tried to reflect the plane strain case with some adaptions.

The situation for the plasticity‐induced crack closure under plane strain condition is significantly different. The crucial point is that there seems to be no source for the plastic wedge, because out‐of‐plane flow is by definition not possible. This point has been clearly expressed by McEvily.^63^ *‘Suppose a crack is growing at a constant load and that the plasticity induced crack closure is occurring in the wake of the crack tip. This closure requires that more material will be behind the tip than it was there before. If the crack grows over some distance, say one meter, where does all the extra material in the wake of the crack come from now?’* For a crack grown over a distance significantly larger than the size of the plastic zone under plane strain conditions with constant stress intensity range or constant load, *ε*_33,_ *ε*_11_ and as consequence *ε*_22_ should be 0 in the wake of the crack tip if the volume during plastic deformation remains constant, which is assumed in all analytical or numerical analyses. In reality, the plasticity of metals and alloys generates dislocations and vacancies. The vacancies induce a volume increase as well as dislocations. The latter is an effect of the nonlinearity in the near core dislocation stress field and the core itself. However, the effect of volume increase due to plastic deformation is small; even in severely plastically deformed metals, the increase is only in the order of 10^−4^ as shown recently.^64^ Therefore, the question remains where does the extra material come from?

Under plane strain conditions and a constant Δ*K* or constant load amplitudes, there is no plastic wedge at large distances behind the crack tip. However, the material in the plastic wake is plastically deformed. It is plastically sheared; this shearing induces a rotation of the volume elements, and as a consequence, a local wedge is formed in the vicinity of the crack tip.^50^, ^51^, ^54^

**Figure 2:**
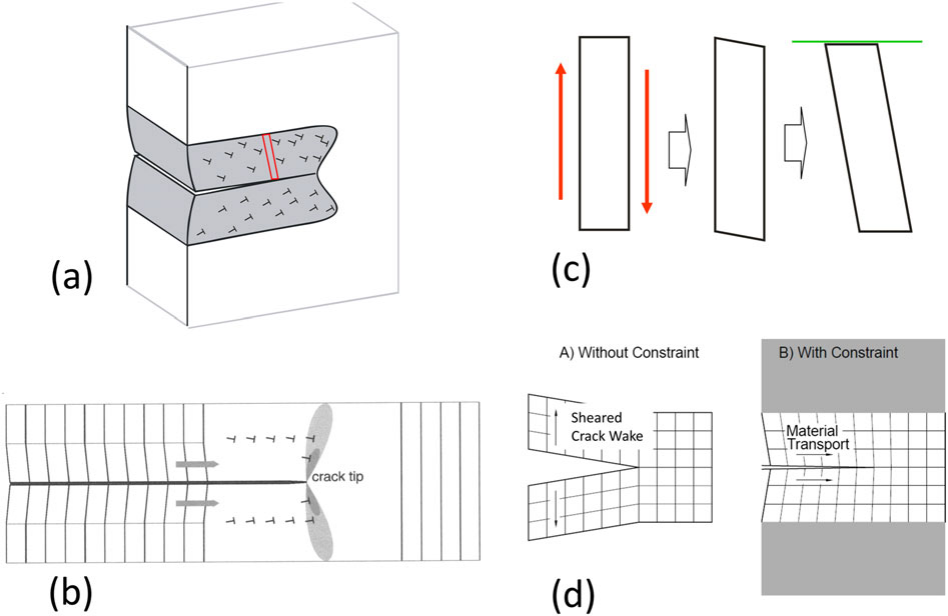
Illustration of the effect of the plastic wake under plane strain and small‐scale yielding conditions on a growing fatigue crack loaded under constant Δ*K*. (a) The plastic shear deformation induces a material transport to the crack tip shown as a consequence of geometrically necessary dislocations in the crack wake. (b) The rotation of the volume elements is visualized from the dislocation point of view: to realize the shear deformation, the geometrically necessary dislocations are arranged for simplicity in a single band and induce the lattice rotation.^59^ (c) Rotational transport of the material from continuum mechanics point of view for a single volume element and (d) for an arrangement of volume elements forming a growing crack.^51^ The shear deformation opens the crack, and without an elastic constraint, this shear deformation would result in a continuous increase of the crack opening displacement with increasing distance from the crack tip. In the unloaded case, the elastic constraint reduces the crack tip opening at lager distances from the crack tip to zero, causes a rotation of the volume elements and closing of the crack in the vicinity of the crack tip. [Colour figure can be viewed at wileyonlinelibrary.com]

There are different ways to visualize this material transfer to the crack tip due to imbedded plastic deformation in the elastic surrounding. For material scientists, the description by geometrically necessary dislocations is the simplest one^50^ (Fig. 2a). Here, the rotation of the volume elements is induced by the ‘small angle grain boundary‐like’ arrangement of the geometrically necessary dislocations in the plastic wake (Fig. 2a & b). From a continuum mechanic point of view (Fig. 2c & d), the constraint shear deformation can explain this material transport to the crack tip.^51^ Another explanation can be given by dislocation shielding considerations.^46^, ^54^, ^65^ Recently, this transfer from the plastic wake to the crack tip has been shown also by a finite element simulation.^66^, ^67^

**Figure 3:**
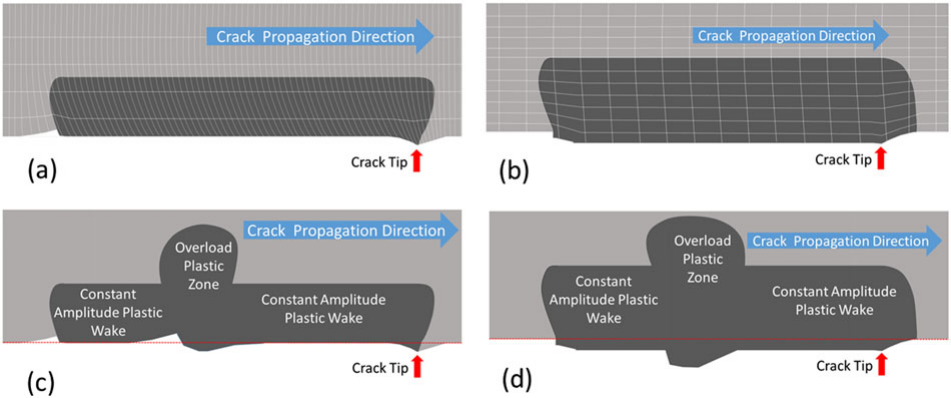
Schematic illustrations of the effect of the plastic deformation of a growing crack illustrated for one specimen half. The resulting displacements are enlarged compared with the size of the plastic zone. (a) Displacement field and the remaining contour, under plane strain conditions; at larger distances from the crack tip, the rotation induced from the shear deformation remains, only near the crack tip a hump is formed with an extension of the size of the monotonic plastic zone. (b) Displacement field and the resulting contour in the plane stress case. Here, the elongation of the volume elements in the plastic wake form a wedge with a constant thickness, even a small effect of shearing takes place (for details, see Ref. [67]). The effect of an overload on the ‘crack’ contour in the case of an overload is depicted for the plane strain (c) and the plane stress (d) cases. [Colour figure can be viewed at wileyonlinelibrary.com]

Figure 3 illustrates schematically the essential difference between the plane stress case (Fig. 3b), where the dominant effect is the elongation of the volume elements in the plastic wake,^67^ whereas in the plane strain case (Fig. 3a), only a local plastic wedge is formed near the crack tip with a length comparable with the size of the plastic zone.^53^ To make the differences clearer in Fig. 3, the ligament of the growing crack containing body is cut along the plane of the crack. As clearly evident from this illustration under plane strain condition, only local wedges or humps on the crack flanks occur, which results from the transfer of the material in the crack propagation direction, for constant and for variable amplitudes, respectively.

### Roughness‐induced crack closure

**Figure 4:**
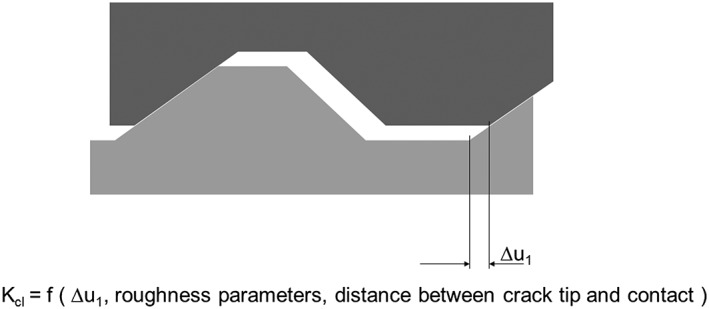
Schematic illustration of roughness‐induced crack closure due to a residual Mode II or Mode III displacement of rough fracture surfaces. The closure stress intensity is determined by the residual displacements *u*_1_ and *u*_3_, the geometry parameters of the rough fracture surfaces and the distance between the crack tip and the contact.

The contact of the fracture surfaces caused by the misfit of the microscopically rough fracture surfaces is denoted as roughness‐induced crack closure. It was first proposed in the early 1980s.^27^, ^28^, ^29^, ^30^, ^31^ The usual explanation of roughness‐induced crack closure is sketched in Fig. 4. At a macroscopic mode I loading, because of microstructural anisotropy and inhomogeneity, a local Modes II or III deformation takes place; this results in a local displacement in the crack propagation direction Δ*u*_1_ or out of plane displacement Δ*u*_3_. In polycrystalline materials, the local Modes II and III deformation vary along the fatigue crack front. In one grain, Δ*u*_1_ and Δ*u*_3_ can be positive, whereas in the neighbouring grains or even in the same grain nearby, an opposite displacement might occur. Hence, this mixed mode‐induced displacement should be only a local phenomenon, because at larger distances behind the crack tip, the residual displacements from the different local crack tip deformation should cancel them out. However, from experimental studies,^68^ it is known that roughness‐induced crack closure can build up over quite large distances, implying that there must be also a far‐field contribution to this closure type.

**Figure 5:**
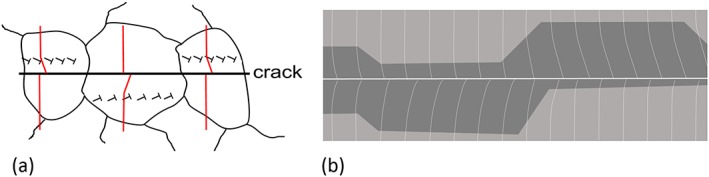
Illustration of the origin of the residual displacement difference Δ*u*_1_ between the fracture surfaces. (a) From the dislocation point of view, the crystal anisotropy in the wake of the crack causes an asymmetric arrangement of the geometrically necessary dislocations.^69^ (b) From the continuum mechanics view, an asymmetric plastic zone develops in the crack wake. The variations in the displacement field on opposite sides of the crack wake are displayed by red (5a) and white (5b) lines. [Colour figure can be viewed at wileyonlinelibrary.com]

**Figure 6:**
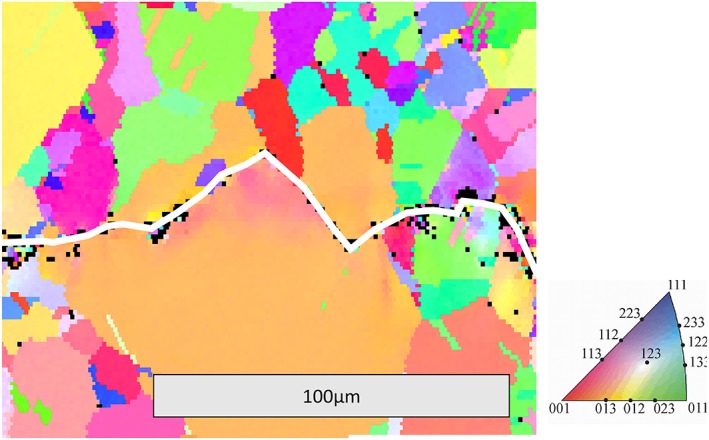
Inverse pole figure maps (crystal orientation map, where the colour code represents the crystal orientation in respect to the macrocrack propagation direction) in the wake of a fatigue crack, grown at Δ*K* = 4.5 MPam^1/2^ at *R* = 0.1 in pure copper. The crack path is marked by a white line. The change of the colour is a measure for the orientation change, that is, the lattice rotation. The difference in the change of the colour (the rotation) on both sides of the crack is responsible for the misfit of the rough fracture surfaces. (For interpretation of the references to color in this figure legend, the reader is referred to the web version of this article.) [Colour figure can be viewed at wileyonlinelibrary.com]

It has been shown that the asymmetry in the deformation at the crack tip generates a misfit of rough fracture surfaces even far away from the crack tip.^69^ The explanation used earlier for the plasticity‐induced crack closure under plane strain condition can be simply adapted to explain the roughness‐induced crack closure. The plasticity‐induced crack closure is explained by a transfer of material to the crack tip; this transfer can be expressed by a displacement of the crack flanks in crack propagation direction Δ*u*_1_. Because of local asymmetric deformation, this displacement varies along the crack propagation direction as shown schematically in Fig. 5. This asymmetry in deformation can be found in all microcrystalline materials and especially at loading conditions near the threshold of the stress intensity range. This asymmetry in the volume rotation is clearly visible from electron backscattering diffraction measurements, which deliver crystal orientation maps near the crack flanks. An example is shown in Fig. 6. From this map, it is evident that

- The orientation of the crystal changes in the vicinity of the crack flanks, as expected from the consideration earlier.
- The amount of the orientation changes is usually different at both sides and changes from grain to grain. Hence, a misfit between the two crack flanks remains.

**Figure 7:**
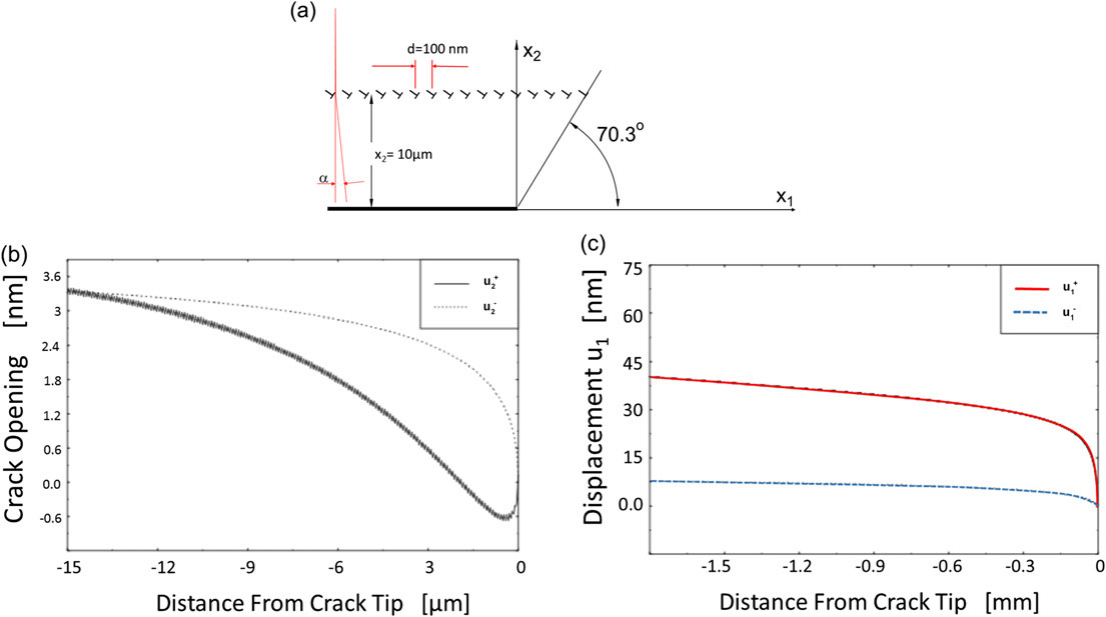
Calculated displacement of the upper and lower crack flank $u_{2}^{+}$, $u_{2}^{-}$ and $u_{1}^{+}$ and $u_{1}^{-}$, respectively. The index +, − refers to the ‘upper’ and ‘lower’ crack flank. The idealized dislocation arrangement in the crack wake is shown in (a). (b) *u*_2_‐displacement contour. A contact of the fracture surface is not taken into account, therefore near the crack tip the two crack flanks overlap. (c) *u*_1_‐displacement contour. After few microns behind the crack tip, the difference between $u_{1}^{+}$ and $u_{1}^{-}$ remains constant.^69^ [Colour figure can be viewed at wileyonlinelibrary.com]

Such experimental results are also reflected by analytical models.^69^ Figure 7 illustrates the displacement contour of a crack in the unloaded state after idealized asymmetric deformation. The case can be described by an exact analytical solution, and for simplicity, all dislocations are arranged in a row at 10 μm distance from the crack flanks to mimic the idealized asymmetric case (Fig. 7a). For the calculation of the fracture surface contour, the contact has not been taken into account. Therefore, crack flanks near the crack tip overlap as shown in Fig. 7b. The size of the overlap region is equal to the assumed size of the monotonic plastic zone. This exact analytical description of the displacement field supports the ideas presented for the plasticity‐induced crack closure under plane strain condition.^50^, ^51^ The displacement difference between the upper and lower crack flank Δ*u*_1_ (Fig. 7c), which in the case of a rough fracture surface would lead to a roughness‐induced crack closure, increases with increasing distance from the crack tip and then remains nearly constant for distances larger than the plastic zone size. A consequence from these analyses is that besides the roughness of the fracture surface,^28^ the asymmetry of the plastic deformation in the plastic wake^69^ is the essential ingredient that controls roughness‐induced crack closure. This is the reason that roughness‐induced crack closure is more pronounced in coarser grained materials near the threshold,^68^, ^70^, ^71^, ^72^ as long as the monotonic plastic zone size is comparable with the grain size or smaller. If the size of the plastic zone is larger than the grain size, that is, the monotonic plastic zone contains many grains, the asymmetric deformation resulting from the crystal anisotropy is reduced.

A simple estimation of the local misfit of fracture surfaces Δ*u*_1_ is given by

(2) $$\Delta u_{1}=\alpha \;x_{2}=\frac{b\;x_{2}}{d_{1}}\;,$$

where *α* is the difference in the rotation angle between the upper and lower plastic wake and *x*_2_ the distance over which this ‘rotation’ takes place. Here, *b* is the length of the Burgers‐vector and *d*_1_ the distance between the dislocations (Fig. 7a). Hence, a large asymmetry in the plastic deformation (large *α*) and a large zone where this asymmetry takes place are important to obtain a high contribution of roughness‐induced crack closure. This seems to be the reason why in ultrafine grained and nano‐crystalline metals, despite a high roughness on the nanoscale, no roughness‐induced crack closure takes place,^73^, ^74^ because according to (2), *x*_2_ is restricted by the grain size. The discussed mechanisms to explain the roughness‐induced crack closure are important for microcrystalline metallic materials in the near threshold regime where the asymmetry in deformation is more pronounced. As mentioned at higher Δ*K*, the asymmetry of the crack tip deformation is reduced. However, fracture surface roughness, crack branching and delamination can result in a local reduction of the stress triaxiality or to a local more plane stress state deformation. This can lead again to an in and out‐of‐plane flow of material and form an additional plastic wedge. This type of mixed roughness and plasticity‐induced crack closure has been discussed elsewhere.^75^

### Oxide‐induced crack closure

The volume of oxides or other corrosion products is typically larger than the volume of the corresponding base material. Hence, the forming of an oxide layer or corrosion debris on the fracture surface can be interpreted as a wedge which is inserted into the crack. Hence, the reduction of the effective stress intensity range due to the oxide‐induced crack closure is straight‐forward. The importance of the crack closure because of fracture surface oxidation has been pointed out by a vast number of researchers (for example, Refs. [32, 33, 34, 35, 36]) at ambient and at elevated temperatures.

The presence of an atmosphere leads to oxidation of freshly formed fracture surfaces, which is often more pronounced in a moist atmosphere.^35^ The thickness of the oxide layer on a freshly formed surface in most metals and alloys is only a few nanometre at ambient temperatures. Because of the fracture surface contact by plasticity and roughness‐induced crack closure, the thickness of the oxide layer can significantly increase. The continuous breaking and reforming during the contact and fretting can lead to a build up of oxide layers in the order of 100 nm. This build up of thick oxide layers on the fracture surface is very pronounced at low *R*‐ratios and very low crack growth rates, that is, near the threshold. In steels, these thick oxide layers formed at such loading condition can easily be seen with the naked eye on the fracture surfaces due to their black appearance.

The oxide‐induced crack closure is important for the explanation of different phenomena near the threshold:

- The effect of environment, for example, the observation of the larger Δ*K*_*th*_ at low *R* ratios in moist air.^35^
- The effect of loading procedure to induce a pre‐crack on the measured Δ*K*_*th*_ 72, 76, 77
- Unexpected load history effects, for example, load amplitudes below the threshold can reduce the growth rates during the following loading.^77^

### Asperity versus wedge‐induced crack closure

Herzberg *et al.* 78 performed fatigue crack growth experiments with single artificial contacts. At relatively large distances from the crack tip (between 10 and 20 mm), shims of different thickness were inserted in the mouth of the opened crack. The reduction in the crack growth rate after introduction of the shims was significantly smaller than expected from the measured *K*_max_−*K*_*op*_ values.

**Figure 8:**
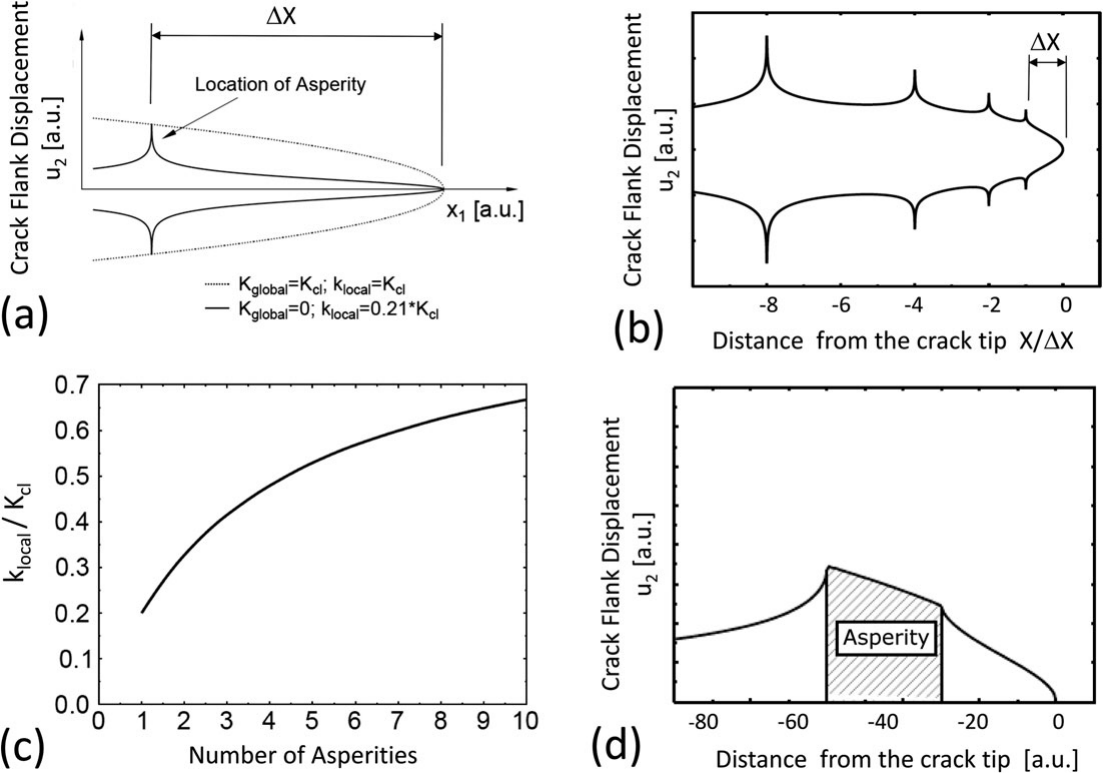
(a) Illustration of the crack contour at the contact load and at zero load; the width of the asperity is 1/1000 of the distance between the asperity and the crack tip. (b) Crack contour of an unloaded crack with four asperities with a width of 1/1000 of the distance between the first asperity and the crack tip. (c) Increase of the local stress intensity with increasing number of equidistant asperities (thickness of the asperities is also 1/1000 of the distance between the first asperity and the crack tip. (d) Effect of the increase of the asperity width on the crack contour. The assumed width of the asperity is equal to the distance from the asperity to the crack tip. The large effect of the width on the local k is visible by comparing (a) and (d); see also Refs. [53, 54].

Riemelmoser *et al.* 54 studied the effect of different asperity geometries on the local stress intensities for linear elastic loaded fatigue cracks. In Fig. 8a, the dotted line shows the contour of the crack at the first contact between the asperity and the crack (*K* = *K*_*cl*_) and the full line when the remote load is zero. It should be noted that in this figure, the scale of the displacement in the *x*_2_ direction is enlarged by a factor of 100. The width of the assumed asperity is 1/1000 of the distance between the crack tip and the contact (the asperity).

From this figure, it is evident that during further unloading (*K* < *K*_*cl*_), a significant deformation, that is, reduction of the local stress intensity occurs. In the unloaded case, the local *k* is not *K*_*cl*_ but only a small portion of *K*_*cl*_. The contribution to the shielding of the crack tip is only 0.21 (*K*_*cl*_−*K*_min_). This is in good agreement with the observation of Herzberg^78^ and the conclusion drawn by Louat.^44^

In Fig. 8b, the effect of multiple asperities, where the contour of an unloaded crack with four small asperities, is plotted. The four asperities are located at 1000, 2000, 4000 and 8000 times the width of the contact, and the height is chosen in such a way that all asperities close at the same stress intensity factor. Figure 8b illustrates that the crack tip shielding by these four very small contacts is significantly larger compared with the single asperity in Fig. 8a. This increase in crack tip shielding with increasing number of contacts is plotted in Fig. 8c. Equidistant, small asperities have a width of 1/1000 of the distance between the crack tip and the first contact. It can be seen that 10 very small contacts can cause a crack tip shielding of about 70% of (*K*_*cl*_−*K*_min_).

A similar behaviour is observed when the width of the contacts is increased. Figure 8d shows the contour of the unloaded crack, where the width of the contact is equal to the distance between the beginning of the contact and the crack tip. The height of the contact is equal to the crack tip opening displacement at the closure stress intensity factor. A comparison with the contour of the very small contact in Fig. 8a clearly shows that the elastic relaxation of the crack tip of the unloaded crack is significantly reduced by the increase of the width of the contact; the shielding of the unloaded crack tip is in this case about 80% of (*K*_*cl*_−*K*_min_).

**Figure 9:**
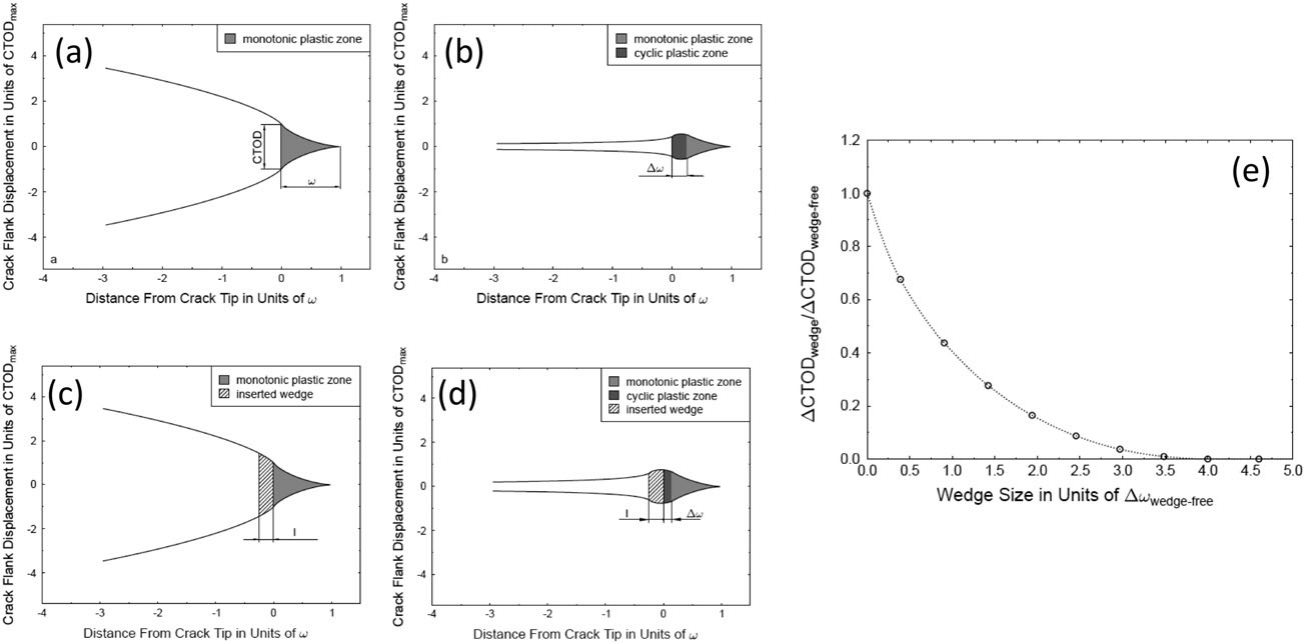
Illustration of the minimum length of a wedge filled into the crack to obtain a certain crack tip shielding. The used Dugdale model is shown in (a) to (d). (a) Crack tip contour of a closure free loaded crack. (b) Configuration of the completely unloaded crack respectively. (c) and (d) present the effect of an inserted wedge on deformation. (e) Decrease of the cyclic crack tip opening displacement with increasing width of the wedge compared with the cyclic crack tip opening displacement without closure.^53^

In contrast to single asperities, a contact that continuously closes from the crack tip, denoted as wedge‐like contact, can completely shield the crack tip. Such wedges‐like contacts are depicted in Fig. 9. However, the wedge must have a certain width, otherwise, the stresses at the contacts will be too high, and the wedge will be ‘plastically compressed’. A simple estimation of the minimum size of a wedge causing a complete shielding, that is, no cyclic plastic deformation should take place, is illustrated in Fig. 9.^51^, ^53^ At maximum load, an artificial wedge with different sizes is inserted immediately behind the crack tip. In order to estimate the minimum length of such a wedge, the Dugdale model^55^ and the McClintock–Rice superposition principle^24^ are used. In the Dugdale model, the plastic zone is replaced by a narrow strip that extends a distance *ω* ahead of the crack tip and is loaded by tractions equal to the yield stress *σ*_*y*_ over a length *x*_min_. During loading, the tractions cause a negative stress intensity factor equal to the stress intensity factor induced by the applied far‐field loading. The cyclic plastic zone of a closure free crack is given in the same way as the monotonic plastic zone, with the difference that the loading parameter is replaced by the stress intensity range and the tractions by twice the yield stress. Figure 9e shows the reduction of the cyclic crack tip opening displacement with increasing size of such an artificial wedge, which also can transfer stresses up to the yield stress of the material. If the deformation during complete unloading should be prevented by a wedge with the minimum width *x*_min_, the tractions induced by the wedge should cause the same stress intensity factor as the far‐field loading at maximum load. Therefore, the calculation of *x*_min_ is identical with the derivation of the monotonic plastic zone size. Hence, the minimum length of the wedge required to induce a complete shielding is equal to the size of the monotonic plastic zone (*x*_min_ = *r*_*pl*_) or 4Δ*r*_*pl*_ of the closure free crack. A consequence of this simple estimation is that one always needs a certain crack extension to build‐up crack closure, not only for the wedge‐like contacts of the plasticity‐induced or oxide‐induced crack closure but also for the roughness‐induced crack closure.

Real cracks at low stress ratios have both type of contacts, asperity‐like and wedge‐like contacts. The aforementioned estimations show that only from the single (or few) asperity‐induced crack closure a determination of Δ*K*_*eff*_ = *K*_max_−*K*_*cl*_ overestimates the effect of crack closure and underestimates the resulting crack propagation rate. However, in most cases, the main problem is not whether the crack tip is shielded completely below *K*_*cl*_ or not, but rather the experimental determination of *K*_*cl*_ itself, because it is very difficult to detect the first fracture surface contact near the crack tip accurately. The length scales involved in this process are quite small as demonstrated in the next section.

### The length scales of fatigue crack propagation

The length scales involved in fatigue crack propagation are the physical crack length *a* (notch depth *D* plus the physical crack length *l* ), the size of monotonic plastic zone *r*_*pl*,_ the size of the cyclic plastic zone Δ*r*_*pl*_, the distances between the fracture surface contacts and crack tip, the width of the contacts, the cyclic crack tip opening displacement, the crack growth rate and the characteristic lengths of a microstructure, which can vary from nanometre to metre, depending on loading condition, the component size, sample size and material.

Assuming small‐scale yielding, which requires that the plastic zone size *r*_*pl*_ is much smaller than the crack length and ligament width, the plastic zone size using the Irwin estimation is

(3) $$r_{\mathrm{pl}}=\frac{K^{2}}{{\pi \sigma }_{y}^{2}}.$$

The cyclic plastic zone size

(4) $$\Delta r_{\mathrm{pl}}=\frac{\Delta K^{2}}{\pi 4\sigma_{y}^{2}}$$

and the cyclic plastic crack tip opening displacement

(5) $$\Delta \text{CTOD}=d_{n}\frac{\Delta K^{2}}{E2\sigma_{y}}.$$

These relations apply when no crack closure occurs, where *E* is the Young's modulus, *σ*_*y*_ is the yield stress and *d*_*n*_ is a dimensionless constant that depends on the stress state, very strongly on the hardening exponent *n* (for a non‐hardening material, it is 1 for plane stress and 0.78 for plane strain, and for a high *n*, it can decrease to about 0.1) and slightly on *σ*_*y*_/*E*; see Refs.[79, 80]. If crack closure takes place, Δ*K* has to be replaced by Δ*K*_*eff*_:

(6) $$\Delta r_{\mathrm{pl}}=\frac{\Delta K_{\mathrm{eff}}^{2}}{\pi 4\sigma_{y}^{2}}$$

and

(7) $$\Delta \text{CTOD}=d_{n}\frac{\Delta K_{\mathrm{eff}}^{2}}{E2\sigma_{y}}$$

The large variation of the length scales is clearly evident from the estimation of the characteristic parameters controlling fatigue crack propagation in Table 1. It has to be noted that the estimation of ΔCTOD near the threshold by Eq. 5 significantly overestimates the value; hence, the variation is even larger.^81^ This indicates that characteristic dimensions vary between nanometre and metre, which causes the difficulties in the experimental and the theoretical analyses of crack closure and fatigue crack propagation phenomena.

**Table 1:** Illustration of the variation of the size of plastic zone, *r*_*pl*_, for stress ratio *R* = 0, the size of the cyclic plastic zone Δ*r*_*pl*_ and cyclic crack tip opening displacement ΔCTOD using the estimations of Eqs 3–5, crack closure for the cyclic values are not taken into account

	Near threshold Δ*K* = 5 MPam^1/2^		High Paris regime Δ*K* = 70 MPam^1/2^	
	Low strength steel *σ*_*y*_ = 250 MPa	High strength steel *σ*_*y*_ = 1800 MPa	Low strength steel *σ*_*y*_ = 250 MPa	High strength steel *σ*_*y*_ = 1800 MPa
*r*_*pl*_	120 μm	2.4 μm	26 mm	0.5 mm
Δ*r*_*pl*_	30 μm	600 nm	6.6 mm	0.12 mm
ΔCTOD	180 nm	25 nm	36 μm	5 μm

### Experimental measurement of crack closure

For the discussion of the experimental evaluation, the vast number of techniques can be divided into three groups:

- compliance techniques
- crack propagation techniques
- non‐mechanical contact measurements.

Each technique has its advantages and specific limitations. However, common for nearly all is the problem of measuring the first contact very near the crack tip, especially at very small load amplitudes.

**Figure 10:**
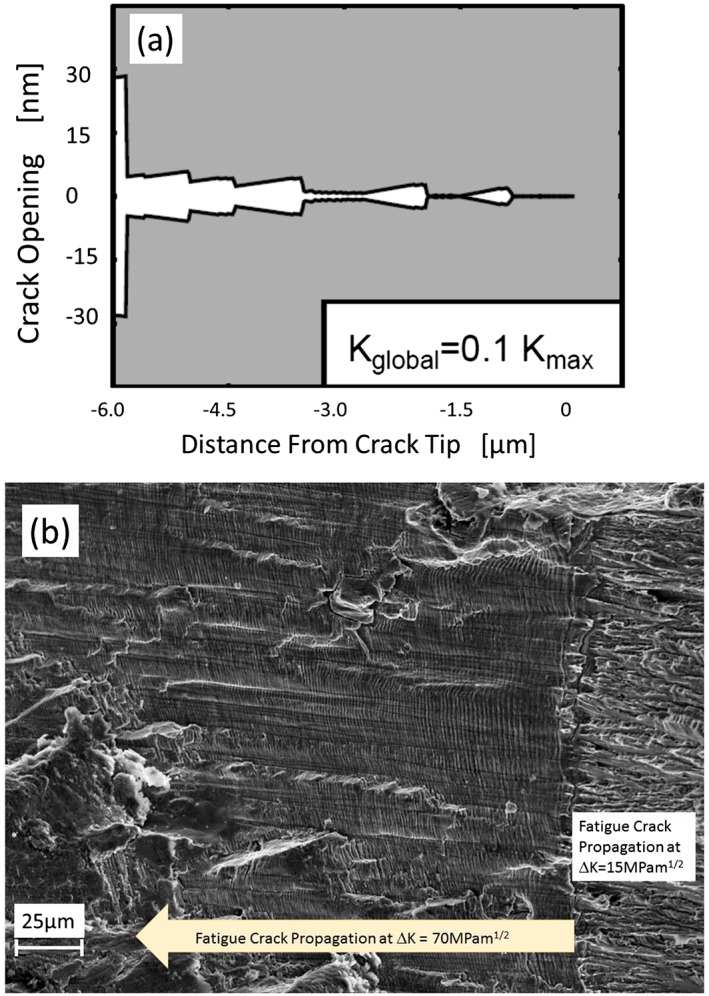
(a) Illustration of the calculated crack contour by discrete dislocation simulation for a loading case near the threshold with a constant load amplitude.^53^ The very small closure over a length of about 1.5 μm has significantly reduced the cyclic crack tip deformation from about 50 nm of the closure free crack at the beginning to about 35 nm after a crack extension of about 6 μm. (b) Fractograph of a fatigue fracture surface generated in the first few hundred cycles at Δ*K* = 70 MPam^1/2^ in a cold worked 316 L austenitic steel. The right side shows the fracture surface of the pre‐crack generated at a small Δ*K*. The reduction of the striation spacing illustrates the fast building up of the plasticity‐induced crack closure under plain strain conditions similar as the simulation in (a), which demonstrated the effect at small Δ*K*. [Colour figure can be viewed at wileyonlinelibrary.com]

Figure 10 shows the calculated contour of a crack at minimum load. The simulated crack is grown at constant Δ*K* over a distance of about 6 μm. The applied discrete dislocation simulation^52^, ^81^ represents an ideal plane strain loading, and the calculated contour of the crack is the sum of the exact analytical displacement solutions of the simulated dislocation arrangement at the minimum load. During the propagation of the crack, the cyclic crack tip opening displacement ΔCTOD decreases from initially about 50 nm to about 35 nm. This continuous reduction of ΔCTOD and the increase of the contact length to about 1.5 μm cause a decrease of the effective stress intensity range Δ*K*_*eff*_ from the initially applied Δ*K* to about 0.8 Δ*K*.^82^ The measurement of crack closure over such a small distance is very difficult and for most experimental techniques impossible to measure; however, for simulation techniques, it also becomes very cumbersome.

How fast plasticity‐induced crack closure under plain strain conditions is built up even for large Δ*K*‐values is demonstrated in Fig. 10b, where the decrease of the striation spacing directly indicates the decrease of Δ*K*_*eff*_.

The compliance technique measures the mechanical response of the contact forces. There are many different techniques to measure the displacement or the local strain:

- mechanical strain or displacement gauges, which can be used in a far‐field or near crack tip arrangement (for example, Refs. [53, 78, 83, 84, 85, 86]),
- interferometric displacement measurement,^87^, ^88^, ^89^
- *in situ* optical microscope or *in situ* scanning electron microscope observation,^89^, ^90^, ^91^, ^92^, ^93^, ^94^
- *in situ* X‐ray tomographic techniques,^95^, ^96^, ^97^
- *in situ* X‐ray stress or strain measurements.^95^, ^97^

Near crack tip techniques are usually more sensitive to measure the fracture surface contacts. However, for some of them, the sensitivity depends strongly on the distance between the sensor and the crack tip and the contact.^86^

**Figure 11:**
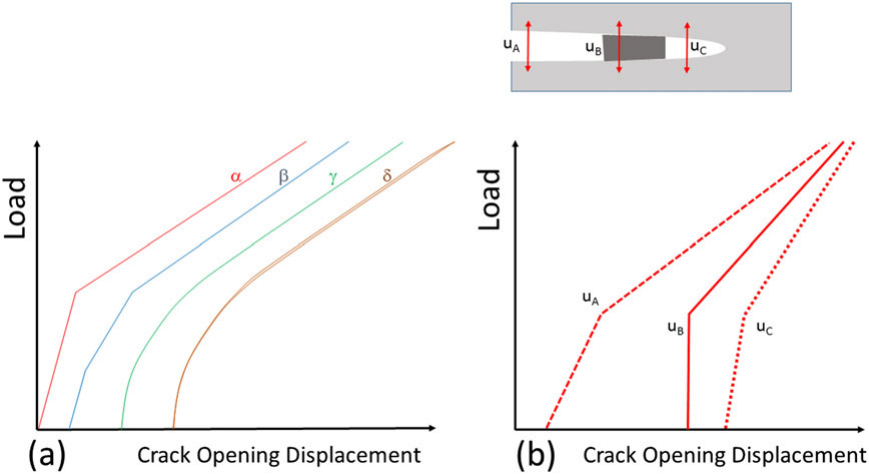
Schematic illustration of load versus crack opening displacement curves.^53^ (a) Effect of different contacts, *α*: single contact at larger distances from the crack tip, *β*: two contacts at different distances behind the crack tip, *γ*: a continuous closing crack and *δ* a continuous closing crack where cyclic plastic deformation and friction between the fracture surfaces takes place. (b) Influence of distance between contact and the position where the displacement is measured. The position of the measurement and the corresponding compliance record are depicted in the schematic. [Colour figure can be viewed at wileyonlinelibrary.com]

The typical load displacement or load strain curves are depicted in Fig. 11. In the case of a single asperity located at larger distances from the crack tip, a well‐pronounced single change of compliance should be observed (Fig. 11a loading case *α*). But real fatigue cracks close continuously from the crack tip, caused by plasticity‐induced crack closure (and maybe by oxide‐induced crack closure) and some asperities caused by roughness‐induced crack closure or corrosion debris, which results in a continuous change of the compliance (Fig. 11a loading case *γ*). In addition, the cyclic plastic deformation and friction of the fracture surfaces result in a small hysteresis of the load displacement or the load strain curve (compare with Fig. 11a loading case *δ*), which makes the determination of *K*_*cl*_ even more difficult. Only a measurement of the strain, the stress or the displacement very near the crack tip permits accurate determination of the fracture surface contact near the crack tip and to determine accurate the effective fatigue crack driving force (Fig. 11b). When the crack closes over larger distances, the evaluation of the *K*_*cl*_ is more straightforward. Hence, in most cases, the problem to determine Δ*K*_*eff*_ is not that *K*_*cl*_ overestimates the real Δ*K*_*eff*_ due to asperity contacts as shown in the last section, but rather the experimental difficulties with the detection of the near crack tip contacts.

**Figure 12:**
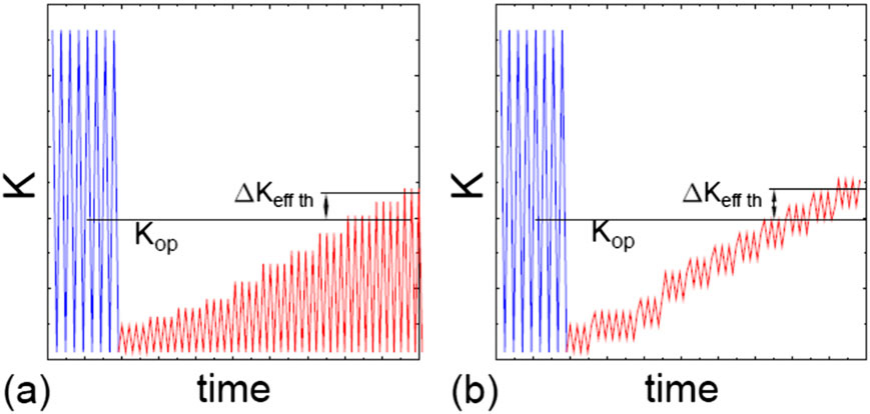
Schematic representation of the loading procedures to measure the crack opening load, *K*_*op*_, after Ref. [89]. [Colour figure can be viewed at wileyonlinelibrary.com]

The second group of techniques, the crack propagation techniques, are based on the assumption that a crack does not propagate when it is closed, and it can only propagate if Δ*K* is larger than Δ*K*_*eff,th*_, and *K*_max_ is larger than the *K*_*cl*_ + Δ*K*_*eff,th*_.^53^, ^98^ Two possible loading procedures to determine *K*_*cl*_ by such method are shown in Fig. 12. In both cases, the crack growth experiments are interrupted at *K*_min_. In the technique depicted in Fig. 12a, at first, a very small Δ*K*, although larger than Δ*K*_*eff,th*_, is applied and increased in steps until the crack starts to propagate. From the Δ*K* value where the crack starts to propagate, the *K*_*cl*_ can be calculated with *K*_*cl*_ = *K*_max_−Δ*K*_*eff,th*_. Another possibility (Fig. 12b) is to apply a small Δ*K*, which is somewhat larger than Δ*K*_*eff,th*_ and increase the mean load until the crack starts to propagate. *K*_*cl*_ can be determined in the same way as in the first technique.

**Figure 13:**
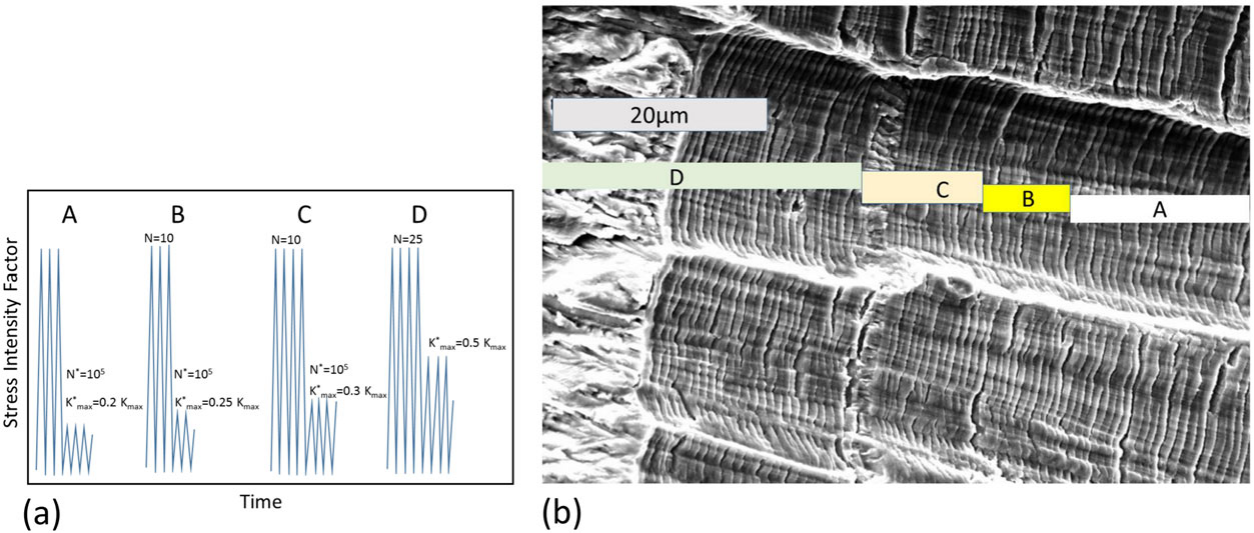
(a) Modified loading procedure to measure the crack opening load with crack propagation technique in materials with a well‐defined striation appearance in fatigue. (b) The resulting fatigue fracture surface of such a test^53^, the first crack extension at the small load is clearly visible at the load block C. [Colour figure can be viewed at wileyonlinelibrary.com]

**Figure 14:**
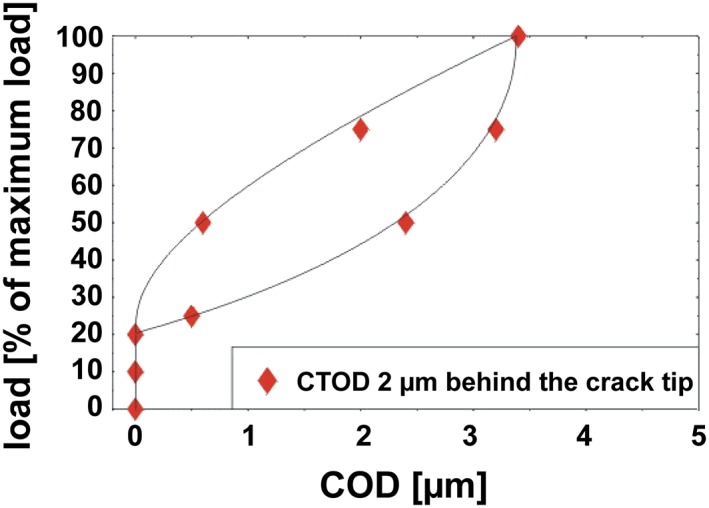
Crack tip opening displacement, measured in the midsection of the thick specimen, 2 μm behind the crack tip determined in a constant Δ*K* test with *R* = 0.05 by means of a special stereophotogrammetric technique.^99^ The measured crack tip opening indicates the closure of the crack at 22% of the maximum load, even at this large Δ*K*‐value where only plasticity‐induced crack closure is active. The experiments were performed with the same material as in Fig. 13, and the determined closure values are in good agreement. [Colour figure can be viewed at wileyonlinelibrary.com]

A result of such somewhat modified experiment is shown in Fig. 13a and b, which was used to determine the crack closure load during constant amplitude loading in an austenitic steel. The difference between the loading procedure shown in Fig. 12b and the used one in Fig. 13a is that after each small load step, few constant load amplitudes of the base loading were applied. The resulting fatigue fracture surface is presented in Fig. 13b. Because of the well‐formed striations generated during the base loading, the load step where the first crack propagation occurred can be clearly detected (load block C), and *K*_*cl*_ can be determined very accurately from the relation *K*_*cl*_ = *K*_max_−Δ*K*_*eff th*_. For comparison, the very near crack tip opening displacement measured with a special compliance technique obtained from direct measurement of the crack tip deformation was used at the same Δ*K* and *R*‐ratio (Fig. 14). The two independent measurements of the closure load are in good agreement. At this relatively large Δ*K*, oxide‐induced and roughness‐induced crack closures are not relevant, and the plane strain conditions were fulfilled, therefore these experiments clearly support the existence of the plasticity‐induced crack closure under plane strain during constant amplitude loading. *K*_*cl*_ is about 0.25 *K*_max_ in the plane strain case, which is in good agreement with the results of the finite element simulations (for example, Ref. [100]). It should be noted that this is significantly smaller than for the plane stress case where *K*_*cl*_ at *R* = 0 is about 0.5 *K*_max_.

The third group of techniques try to measure the occurrence of a contact by optical,^86^, ^87^, ^88^, ^89^ acoustic^101^ or electrical methods.^102^ In transparent materials, optical technics can be used to determine both the change of crack tip opening displacement during the load cycle by a Newton interference method and determine the contact area and location.^88^, ^89^ The acoustic methods use the change of transmission or reflection of acoustic waves from closing crack flanks.^101^ The advantage of these methods is that they permit a through thickness evaluation of the closure process. Furthermore, the acoustic emission when the crack faces come into contact has been used to determine the closure load.

The electrical resistivity of the oxide layer on the fracture surface of most metals and alloys is very high; therefore, the change of the potential drop during the fracture surface contact is very small. Only for fatigue crack propagation in high or ultra‐high vacuum the closure of the fracture surface contacts significantly effects the potential drop.^102^ But few friction contacts of the fracture surface causes potential drop changes during the whole loading range, which makes the determination of the mechanically effective contacts difficult. Similar problems arise in the use of the acoustic emission method.

### Prediction of crack closure

For materials where the fatigue crack propagation is solely governed by the cyclic plastic deformation, which is typical for ductile materials and for loading significantly below the fracture toughness, the knowledge of the effective stress intensity range or the effective Δ*J* should be sufficient for the prediction of the fatigue crack growth rate.

Therefore, the description of the

- mean stress or load ratio effects,
- the effects of load history, that is, the effect of overloads, underloads and variable amplitude loading,
- and the effect of physically short cracks

is fundamentally related to the changes of the crack closure. The prediction of the crack closure load along with the knowledge of *da*/*dN* versus Δ*K*_*eff*_ are the essential ingredients for an accurate lifetime calculation.

The different approaches to predict crack closure can be divided into

- strip yield models,
- elastic plastic finite element analyses and
- analytical or half‐analytical models, which are partly physically or empirically related.

The prediction of crack closure and the lifetime prediction in general under variable loading is a very broad field, and the goal of this section is not to review all of the studies. Here, only the possibilities and limitations of the different groups are shortly summarized, and some important open questions will be pointed out.

The strip yield models are based on the Dugdale model. The first models of this type were introduced by Führing and Seeger,^57^ Newman^58^, ^103^ and de Koning.^59^ The numerical simulation uses thin strip elements along the crack line. These strips in the plastic zone ahead of the crack are intact and carry both tension and compressive stresses. The elements in the wake are broken and can only carry compressive stresses during the loading phase when they are in contact. Outside the strip, the material is perfectly elastic, like in the Dugdale model. During unloading, the contact stresses are computed. The displacement of the strip (height of the bar elements which form the plastic wedge) is adapted if the stress is larger than the yield stress. The main difference between the models is how the constraint is taken into account in order to mimic plane stress or the plane strain conditions; for details, see Refs. [62, 66, 100]. The strip yield model is a good physical approximation for the description of the plasticity‐induced crack closure under plane stress conditions. However, for fatigue crack propagation under plane strain conditions, it does not reflect the real physical phenomena (see again Fig. 2). By varying the constraint parameters in the model, a somewhat thinner wedge can be obtained to mimic a more local wedge, which is typical of the plane strain case (Fig. 3a).

Numerous finite element or boundary element analyses have been performed to simulate fatigue crack growth (for example, Refs. [103, 104, 105, 106, 107, 108, 109, 110, 111, 112, 113, 114, 115]). Most of these studies were conducted to obtain a basic understanding of the crack closure mechanisms and how the crack tip stress and strain fields are influenced by the plastic deformation in the plastic zone and the wake of the crack tip. The majority of these studies were performed either under plane stress or plane strain conditions.

Only a relatively small number of three‐dimensional finite element analyses (for example, Refs. [104, 105, 106] of fatigue crack propagation have been performed. The vast majority of these studies focused on plasticity‐induced crack closure, only a few attempts have been made to simulate roughness‐induced and oxide‐induced crack closures.^108^, ^109^ One of the most crucial points is the element type and mesh size,^110^, ^111^, ^112^, ^113^, ^114^, ^115^, ^116^, ^117^ which is especially significant for the plane strain case, where the plastic ‘wedge’ is very short. The mesh size is critical and may be one of the main reasons for the discrepancies observed under plane strain conditions. Because of the extended wedge under plane stress, this point is not as crucial. Most of the studies are performed for a specific cyclic and monotonic material behaviour, and only very few studies consider a monotonic and cyclic hardening or softening^118^, ^119^, ^120^ as well as ratchetting.^121^ Only the effect of the ratio of the loading stress to yield stress has been investigated in detail (for example, Refs. [100, 105, 120]). The general trend of these analyses shows that with an increasing ratio of applied stress to yield stress, the ratio of the closure stress to applied stress decreases. Only in the well‐defined small‐scale yielding regime under constant amplitude loading the closure stress intensity factor depends only on the stress ratio. This clearly demonstrates that crack closure is a consequence of the encapsulated plastic deformation in an elastic surrounding.

The finite element method is the best tool to study all material parameters and loading parameters as well as the 3D effects that influence the plasticity‐induced crack closure. However, more research is needed to draw a clear quantitative picture of the load, load interaction effects and the influence of the plastic behaviour of the material on plasticity‐induced crack closure, which is the only effect that should be described without assumptions. With such basic knowledge, other, not as cumbersome, tools such as the strip yield method or other more analytical tools could be calibrated.

In the third group, we have summarized a large number of methods that usually do not examine the details of the closing and opening phenomena of the crack or the details of this effect on the cyclic plastic deformation. Some of them are physically based, others are a purely empirical description by fitting of experimental observations, like the NASGRO equation (for example, Refs. [122, 123, 124]). By combining empirical relations such as the Wheeler model^124^ with the closure behaviour, or using dislocation shielding concepts, one could attempt to predict the load history effects (for example, Ref. [65]).

As already mentioned previously, from a physical point of view, only the plasticity‐induced crack closure can be predicted without questionable assumptions. For example, a physical prediction of the roughness‐induced crack closure would require a detailed description of the fatigue crack propagation by crystal plasticity over a large growing distance compared with the characteristic microstructural dimensions and an assumption of the roughness details of fracture surface.

A description of the oxide‐induced crack closure would require information of the growth of the oxide layer or asperities as a function the contact type, the friction, the contact load, the environment and the evolution of the oxide growth process during the cyclic loading. Most of these details required for the prediction of the roughness‐induced crack closure and the oxide‐induced crack closure are unknown.

**Figure 15:**
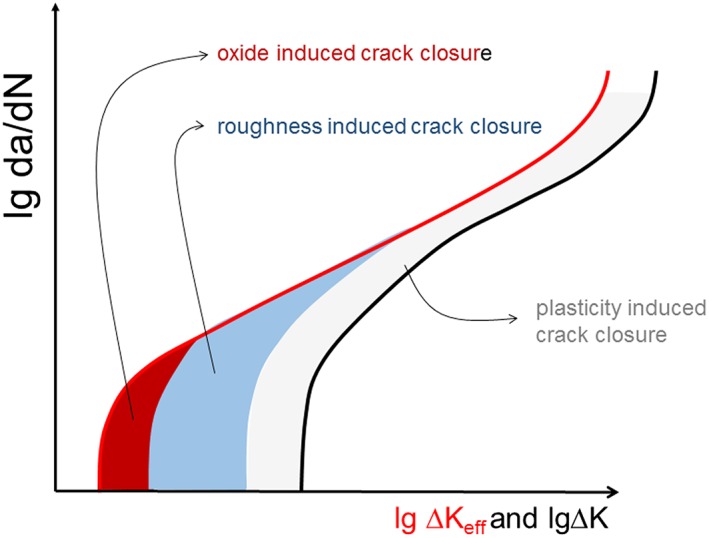
Schematic illustration of the effect of oxide‐induced, roughness‐induced and plasticity‐induced crack closures on the fatigue crack propagation behaviour (*da*/*dN* versus Δ*K*) in ductile materials, where Δ*K*_*eff*_ controls the fatigue crack growth rate. [Colour figure can be viewed at wileyonlinelibrary.com]

Therefore, some of the empirical models are useful because they incorporate all three closure mechanisms. But even for the simple constant amplitude loading (see the schematic in Fig. 15), a simple description for the oxide‐induced and roughness‐induced crack closures as a function of Δ*K*, the stress ratio *R* and the environment does not exist.

**Figure 16:**
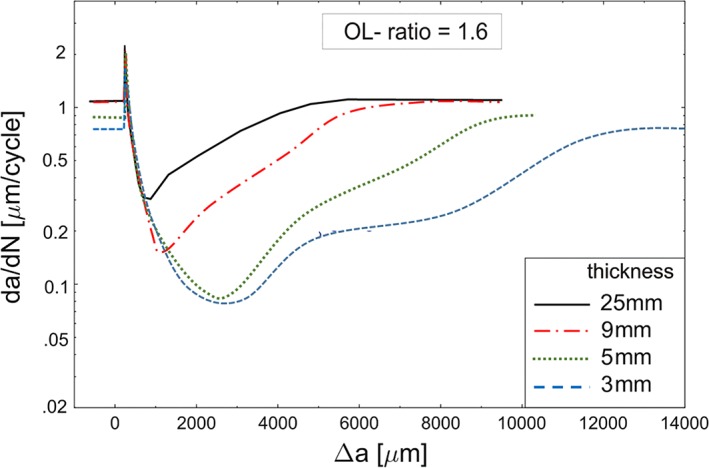
Effect of specimen thickness (i.e. stress state in the plastic zones) on the retardation effect after a single overload.^99^ The base load amplitude was 70 MPa·m^1/2^, the effect of stress state, is even visible in the steady state fatigue crack propagation rate. The crack propagation rate was determined from the striation spacing. [Colour figure can be viewed at wileyonlinelibrary.com]

Even for the most simple case of load interaction of a single overload in the mid Paris regime, where roughness‐induced and oxide‐induced crack closures are insignificant, the experimentally observed retardation effects cannot be fully predicted by the present models. Figure 16 shows the experimental results of overload experiments on samples with very different thicknesses.^99^ The strong thickness effect is evident. In the 25‐mm‐thick sample, the overload plastic zone is dominated by the plane strain state. In the 9‐mm‐thick sample, the overload plastic zone is mainly dominated by the plane stress state, whereas the cyclic plastic zone during the unloading from the overload and the monotonic plastic zone during the base loading are dominated by plane strain. In the thinnest sample (3 mm), only the cyclic plastic zone during base loading is dominated by the plane strain state, the others are dominated by the plane stress state. These experimental results and estimations of the deformation conditions, which have to be taken into account for an accurate prediction of the propagation behaviour, reflect the complexity of this simple loading case. Only a careful 3D finite element simulation of the different loading cases could deliver a description of the observed phenomena.

Besides the loading effect, the influence of how the plastic properties of the material as cyclic and monotonic hardening or softening affect this phenomena is not analysed in detail. Despite the importance of the prediction of crack closure, it is surprising that such basic open questions are not satisfactorily solved.^120^, ^125^, ^126^, ^127^, ^128^

### Crack closure versus compressive residual stress

It is well known that residual stresses significantly affect the fatigue crack propagation behaviour.^1^, ^30^, ^129^, ^130^, ^131^ Macroscopic residual stresses can be considered as an additional applied static loading, and they change locally the stress ratio. In special cases, the effect of the residual stresses on the fatigue crack propagation rate can be used to estimate these local variations of the residual stresses.^131^

Because of plastic deformation in front of the crack in an unloaded sample or component, always a characteristic residual stress field remains, depending on the applied load history. During changes of the load amplitude, the residual stress field changes. There are several authors which try to describe the load interaction effects solely by changes of the residual stresses in front of the crack. In principle, such approaches could be used to take into account all effects – stress ratio, load interaction or physical short crack effects. However, a detailed description of the residual stresses including the contact stresses at the minimum load in each load cycle should be incorporated. Such approach is more or less equivalent with a detailed description of the cyclic plastic deformation in front of the crack tip in each cycle, which is very difficult. In order to avoid the cumbersome calculations, simple estimations are introduced, which can reflect some observations during variable amplitude loading.^132^, ^133^, ^134^ However, several details cannot be described with such simplifications of the effect of the residual stresses in front of the crack. For single overload, the arising problems will be shortly discussed.

A sufficient large overload induces a ‘residual blunting’ that opens the crack (for example, Ref. [99]. Because of the additional plastic deformation induced by the overload, a larger zone with compressive residual stresses develops. These larger compressive residual stresses are used to explain the retardation effect.^132^, ^133^, ^134^ The largest residual stresses in front of the crack are present immediately after the overload, hence the largest reduction of the crack propagation rate should be observed immediately after the overload.

However, in the first few cycles after the overload, cracks propagate faster than before the overload (Fig. 16), and then the crack growth rate decreases below the growth rate of the base amplitude loading; this phenomenon is usually called the delayed retardation. Because of the more extensive blunting at the overload, the fracture surface contact is reduced or disappears completely, which causes the initial increase of the crack propagation rate. On the new fatigue fracture surface generated in the loading cycles after the overload, the fracture surfaces come again into contact, and compressive stresses are transferred; this leads to a continuous decrease of the growth rate.^99^ Because of larger plastic deformation at the overload, the contact stresses and the region where this contact stresses act are larger than during constant amplitude loading. In other words, the closure load is larger than during the base loading, or the effective stress intensity range is smaller than during base amplitude loading. If not only the residual stresses at the minimum load in front of the crack but also the fracture surface contacts were taken into account, the residual stress concept would allow to describe the delayed retardation and would be more or less equivalent to the closure concept.

### Physically short crack effect

Short cracks grow often significantly faster than long cracks and can propagate below the long crack threshold stress intensity range. This was first presented by Pearson.^40^ A vast number of papers and several conferences are devoted to this important problem. In order to understand the different phenomena, it is very helpful to divide the different short cracks^41^, ^135^ into classes.

- For *microstructurally short cracks,* the crack size is comparable or smaller than the characteristic microstructural dimension, such as the grain size or the spacing of different phases in composites.
- For *mechanically short cracks*, the plastic zone size is in the order of the crack length or larger, so that small‐scale yielding is not fulfilled, which is often the case during low and high cycle fatigue testing of smooth specimens. How crack closure changes for this class of cracks will be discussed in the next chapter.
- Fatigue cracks or flaws in components where small‐scale yielding is applicable but crack tip shielding mechanisms not fully developed are denoted as *physically short cracks* or *extrinsically short cracks*.
- In aggressive environments, cracks below a certain crack size behave differently and are termed *chemically short cracks*. This is caused not only by a change of the chemical transport in the crack but also by a change of asperity‐induced crack closure.

Cracks emanating from small flaws or sharp notches usually are initially open over the full load range. With crack extension, the newly generated fatigue fracture surface can come into contact, and the closure stress intensity factor increases. This corresponds to the physically short crack case.

**Figure 17:**
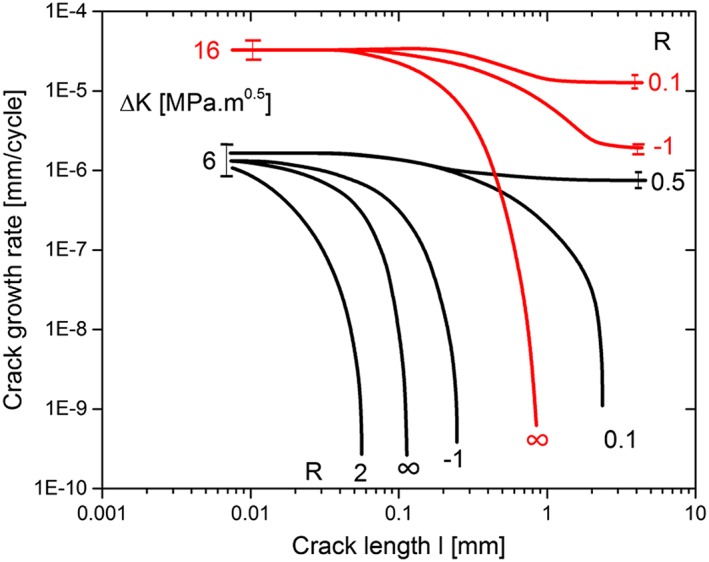
Illustration of the fatigue crack propagation behaviour in deep sharp notched specimens (crack‐like notches) as a function of crack length measured from the notch root at constant Δ*K* = 16 and 6 MPa·m^1/2^ and different stress ratios. *R* = ∞ means pure cyclic compression. The experiments were performed with pure iron (ARMCO iron). For further details, see Ref. [136]. [Colour figure can be viewed at wileyonlinelibrary.com]

Figure 17 shows the fatigue crack propagation behaviour on very sharp deep notches, in ARMCO iron (technically pure Fe). The experiments are performed at constant Δ*K* at different stress ratios. At stress ratios between 0.5 and 1,^136^ the crack growth rate is independent of the crack length measured from the notch root. For smaller stress ratios, the growth rate decreases till it either becomes constant or drops towards zero. For very short crack extension, the crack growth rate is independent of stress ratio and independent whether the experiment is performed in remote compression loading (*R* = 2;∞), tension–compression (*R* = −1) or in tension loading (*R* = 0.1; 0.5), that is, the effective driving force is always Δ*K* or *K*_*cl*_ = *K*_min_. These experimental results clearly show that only the cycle deformation determines the crack propagation behaviour, or in other words, the contribution of the monotonic deformation can be ignored in this ductile materials at these relatively low Δ*K* values. A similar behaviour can be also seen on the fractograph in Fig. 10b, where the initial decrease of the growth rate due to the building up of crack closure at the beginning loading block with a large load amplitude is clearly visible.

**Figure 18:**
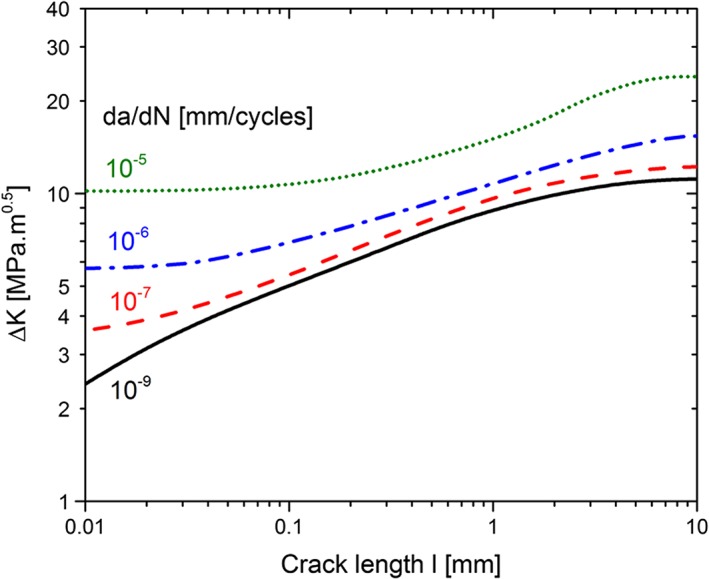
*R*‐curve for fatigue crack propagation of ARMCO iron with a grain size of about 70 μm at a stress ratio of 0.1. The data are obtained from constant Δ*K* tests on deep sharp notched specimens performed at different Δ*K*‐values.^137^ [Colour figure can be viewed at wileyonlinelibrary.com]

The reduced effect of crack closure in physically short cracks is responsible for the abnormal fast crack growth and the crack propagation below the long crack threshold. Therefore, the crack growth of physically short cracks could be either described by the increase of crack closure or can be interpreted as an *R*‐curve behaviour of fatigue cracks. In fracture mechanics, the increase of the fracture resistance is denoted as an *R*‐curve behaviour. Such *R*‐curves are depicted in Fig. 18.^137^ It shows the necessary Δ*K* to obtain a certain crack propagation rate. Such *R*‐curves for different growth rates are unusual, because they are difficult to measure. An exception is the *R*‐curve for the threshold of stress intensity range, which represents the *R*‐curve for *da*/*dN* = 0.^42^, ^72^, ^75^, ^137^, ^138^, ^139^

**Figure 19:**
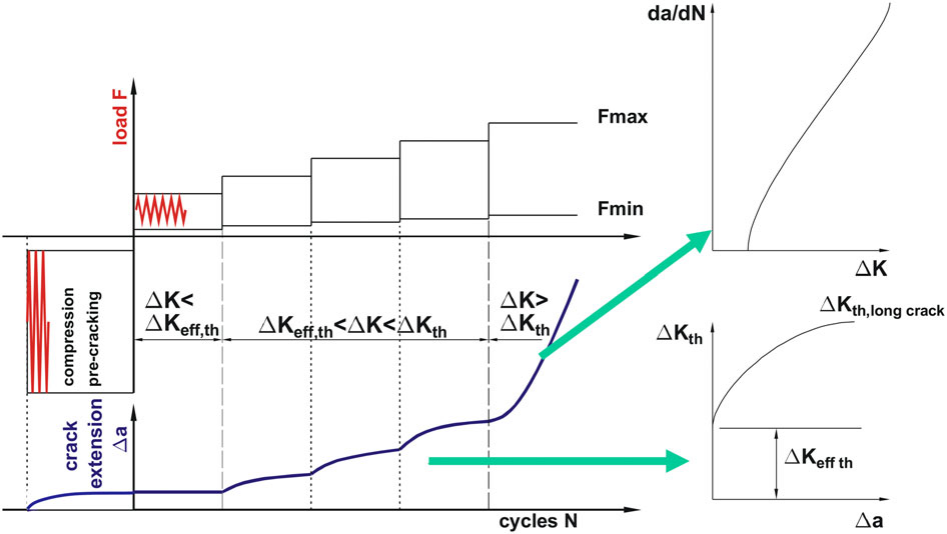
Illustration of stepwise increasing load amplitude test to determine the *R*‐curve of the threshold of stress intensity range, the long crack threshold and the long crack *da*/*dN* versus Δ*K* behaviour. The loading procedure, the resulting crack extension as a function of number of cycles, the resulting *R*‐curve for Δ*K*_*th*_ and *da*/*dN* versus Δ*K* curve are schematically illustrated.^139^ [Colour figure can be viewed at wileyonlinelibrary.com]

A relatively simple technique to measure such *R*‐curves for Δ*K*_*th*_ is the stepwise increasing load amplitude test on deep notched specimens.^139^ The requirement for such measurement is to produce a pre‐crack, which is open during the full load amplitude and contains no or at least very small residual stresses in front of the crack. A load procedure to measure such *R*‐curve for Δ*K*_*th*_ is shown in Fig. 19. In this case, the pre‐crack is generated in pure compression. The load should be as small as possible (hence a sharp notch is required), and the number of cycles should be relatively large to reduce residual stresses to a minimum. The opening stress intensity is in such case about the maximum stress intensity factor of the pre‐cracking (which is zero or a small negative value) minus the effective threshold of stress intensity range. If possible, an annealing to reduce further the residual stress is an option.

In the stepwise increasing load amplitude test, to measure the *R*‐curve for the threshold, one starts with a very small load amplitude. If Δ*K* is smaller than Δ*K*_*eff,th*_, no crack propagation will be observed. If Δ*K* is larger than Δ*K*_*eff,th*_ but smaller than the long crack threshold Δ*K*_*th*_, the crack will start to propagate till the effect of the closure crack reaches a value that Δ*K*_*eff*_ ≤ Δ*K*_*eff,th*_.

If the load amplitude corresponds to a Δ*K* larger than Δ*K*_*th*_, the crack will not stop to propagate, and the standard *da*/*dN* versus Δ*K* behaviour can be determined from the crack length versus the number of cycles. Δ*K*_*eff,th*_ is between the Δ*K* values of the load blocks where no crack propagation and the first crack propagation is observed which might be difficult to measure, especially in high strength materials, where the *R*‐curve for Δ*K*_*th*_ is often very steep. The long crack threshold Δ*K*_*th*_ lies between the Δ*K* values of the load blocks where the crack stops for the last time and the load block where the crack does not stop anymore. From the stopping crack extension and corresponding Δ*K*, the *R*‐curve for Δ*K*_*th*_ can be determined. The increase of Δ*K*_*th*_ in such *R*‐curve can be interpreted as the increase of crack closure stress intensity as a function of the crack length at the threshold of stress intensity range (for details, see Ref. [139]).

The short crack behaviour is especially pronounced at low and negative *R*‐ratios, because there, the decrease of Δ*K*_*eff*_ with increasing crack length is very pronounced. Furthermore, it should be noted that for negative *R*‐ratios, the *K*_*cl*_ (or the closure value) may also depend on the applied minimum stress and not solely on the *R*‐ratio as presented in Fig. 17. Especially pronounced is this effect when the negative applied stresses become into the order of the yield stress,^140^ because due to the high contact stresses, the contacts may deform plastically, and the closure value decreases, that is, Δ*K*_*eff*_ increases.

### From small‐scale yielding to large‐scale yielding

For long fatigue cracks under small‐scale yielding conditions, the closure load is always a tensile load. Only immediately after an overload or in the case of large compressive contribution with compressive stresses near the yield stress the opening load or closure load is near zero load.^141^ What happens when the small‐scale yielding condition is not fulfilled? This question was treated at first by Dowling and Iyyer.^142^

**Figure 20:**
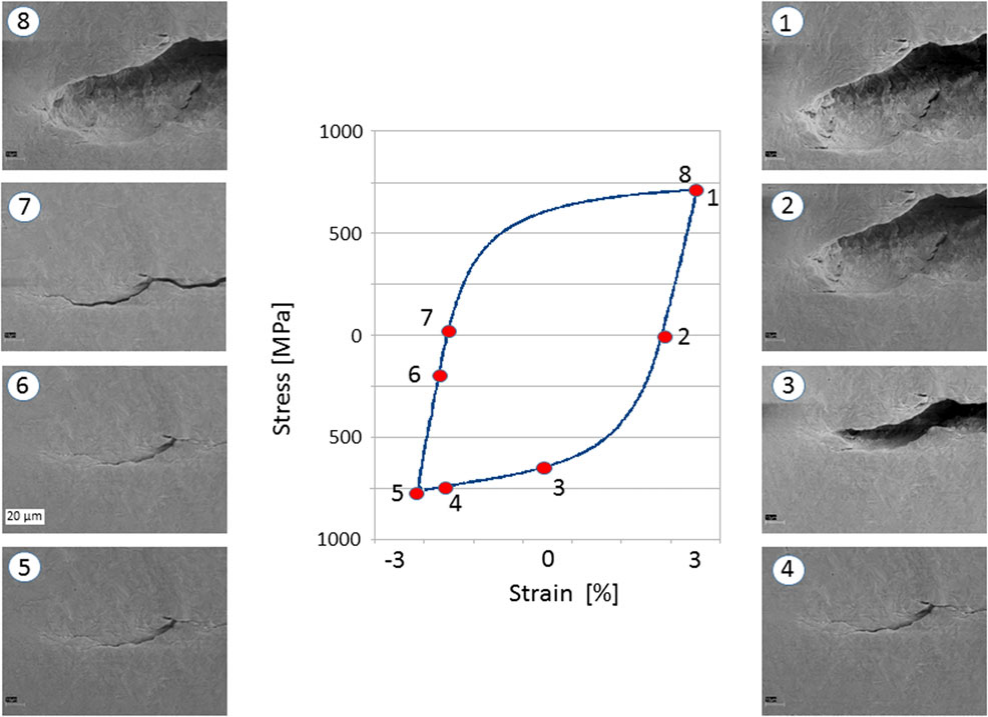
Illustration of the crack closure under low cycle fatigue loading (full‐scale yielding conditions). Scanning electron microscope images are taken at different positions of the stress–strain hysteresis curve.^91^ In this graph, the stresses and strains refer to the uncracked cross‐section of the specimen. [Colour figure can be viewed at wileyonlinelibrary.com]

Figure 20 shows this extreme behaviour of the short crack under low cycle fatigue loading. The experiment at constant strain amplitude was performed in a scanning electron microscope^91^ (SEM). The obtained stress–strain behaviour and the SEM images of the near crack tip regime at the different loads are depicted in Fig. 20. Because of the large strain amplitude, a typical hysteresis of the stress–strain curve is observed.

Figure 14 showed also the crack tip opening displacement measured directly at the crack tip of the same austenitic steel (however in a cold worked state) of a long crack under small‐scale yielding conditions with comparable cyclic crack tip opening displacement and comparable *da*/*dN*; for experimental details, see Ref. [9].

The essential differences between small‐scale yielding and low cycle fatigue are as follows:

- Under small‐scale yielding and constant amplitude loading, the crack closure and crack tip opening loads are about equal, but under low cycle fatigue (LCF) loading, the crack closure and the crack opening stresses are very different. The crack closes near the minimum stress (Position 4) and opens near the transition from compression to tension (between Positions 6 and 7).
- For loading with constant strain amplitude, the strain where the crack closes and opens is equal. Hence, for LCF, it makes more sense to define an effective strain amplitude and not an effective load or stress amplitude. The effective strain amplitude during constant strain amplitude loading is about

$$\Delta \varepsilon_{\mathrm{eff}}=\Delta \varepsilon -\varepsilon_{y}$$

where *ε*_*y*_ is the elastic component of the strain amplitude Δ*ε*_*el*_/2. In principle, under small‐scale yielding, a definition of an effective strain amplitude is also useful. Because of the linear relation between stress and strain, it is not important how one evaluates the effective crack driving force by Δ*σ*_*eff*_ or Δ*ε*_*eff*_.

**Figure 21:**
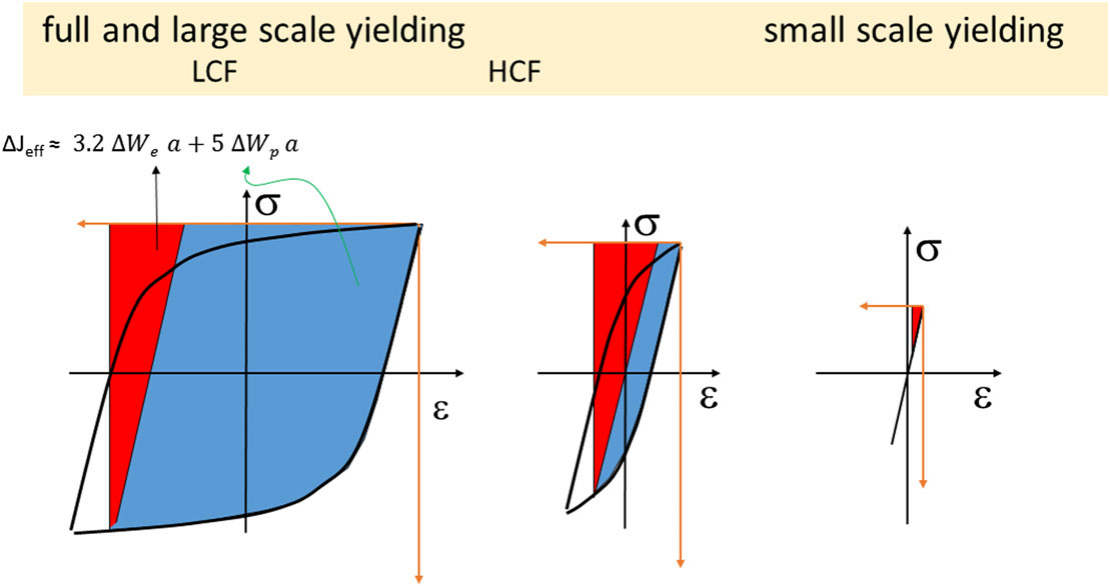
Illustration of the effect of crack closure on the effective crack driving force in the different loading regimes of fatigue crack propagation, from full‐scale yielding to small‐scale yielding.^91^ [Colour figure can be viewed at wileyonlinelibrary.com]

**Figure 22:**
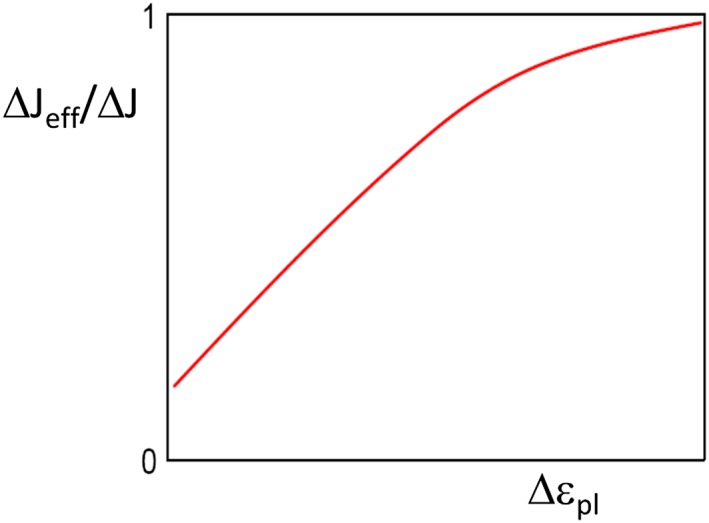
Effect of crack closure (compressive load‐induced closure) on the effective crack driving force in the low and high fatigue regimes.^91^ [Colour figure can be viewed at wileyonlinelibrary.com]

The consequence of this change of the closure behaviour from the LCF to the small‐scale yielding case is schematically depicted in Fig. 21. At large plastic strain amplitudes, considering a strain‐controlled experiment, in metallic materials, usually a stress ratio of −1 develops, independent of the starting condition, hence the schematic Fig. 21 is drawn for a stress ratio of −1. It is evident from this figure that the effect of crack closure is small at a very large strain amplitude and increases with decreasing strain amplitude. In other words, the ratio of the effective driving force to the macroscopic applied crack driving force decreases significantly with decreasing plastic strain amplitude (Fig. 22). This is especially pronounced when the plastic strain amplitude becomes very small.

The term crack closure has been originally introduced to incorporate that growing fatigue cracks are not open during the complete tension part of the load amplitude. During LCF loading, the term crack closure is used here somewhat more general, if a crack is closed over a certain part of the strain amplitude. But we have already seen that physically short cracks can be open also during the compression phase of the load amplitude; however, the closure load in this case increases till it reaches the long crack closure value, which is in the tension regime. That is different in the LCF regime, where the effective strain amplitude remains relatively constant independent of the crack extension. The crack closure during LCF loading is simply a consequence of the elastic load bearing capacity of the crack wake. Cyclic creep may shift the opening load into the compression regime.

The origin of the crack closure in the LCF regime is therefore different to the crack closure under small‐scale yielding condition. Because of the high stresses on the crack flanks, which can lead to a plastic deformation of the crack wake, the roughness‐induced and oxide‐induced crack closures are of minor importance in this case. A change of the loading amplitude from the LCF to the high cycle fatigue regime and the small‐scale yielding regime will change the closure mechanisms from the simple compression load‐induced crack closure (elastic load bearing of the crack wake) to the plasticity‐induced, roughness‐induced and oxide‐induced crack closures.

## Conclusions

In ductile materials, crack closure is essential to explain the effects of stress ratio, short cracks and load history on fatigue crack propagation. The origin of the plasticity‐induced, roughness‐induced and oxide‐induced crack closures and their consequences for the experimental verification and the prediction are reviewed.

- The plasticity‐induced crack closure under plane stress and under plane strain conditions require different explanations. For constant amplitude loading, the plasticity‐induced crack closure under plane stress conditions can be interpreted as a wedge filled into the crack, induced by the out‐of‐plane flow in the plastic zone. In the plane strain case, only a local wedge near the crack tip develops as a consequence of the plastic shear deformation in the plastic wake of the crack. The size of this wedge is in the order of the size of the plastic zone.
- Roughness‐induced crack closure is a consequence of the asymmetric in‐plane and out‐of‐plane displacements of the crack flanks induced by asymmetric deformation of the plastic wake.
- Asperity‐induced crack closure does not completely shield the crack tip after the first contact because of elastic relaxation of the crack contour between the crack tip and the asperity during the unloading phase. For a large number of asperity contacts, this reduction of crack tip shielding is small, which means that a large number of asperities behaves like a wedge and is well approximated by Δ*K*_*eff*_ = *K*_max_−*K*_*cl*_.
- Crack tip shielding by crack closure requires a certain width of the fracture surface contact. However, this length can be quite small. A wedge‐like contact behind the crack tip of one‐fourth of the cyclic plastic zone can induce significant reduction of Δ*K*_*eff*_, and a significant crack tip shielding.
- This relatively small necessary contact width causes the problems in the experimental verification of crack closure by far‐field closure measurement techniques, and also, simulations require a very detailed analysis of the near crack tip deformation.
- Plasticity‐induced crack closure is in principle predictable; however, there are many open questions in the exact prediction, especially the 3D effects, transition from the plane strain to the plane stress case, of the effect of cyclic and monotonic softening or hardening on the closure behaviour of the crack.
- Prediction of roughness‐induced and oxide‐induced crack closures requires extensive additional experimental and theoretical studies.
- Crack closure is not only important under small‐scale yielding conditions; it plays a crucial role under large‐scale yielding as well. In the transition regime, the crack closure load decreases and can decrease into the compression regime of the load amplitude for high and low cyclic fatigue loadings.
